# Rare Variants in Neurodegeneration Associated Genes Revealed by Targeted Panel Sequencing in a German ALS Cohort

**DOI:** 10.3389/fnmol.2016.00092

**Published:** 2016-10-13

**Authors:** Stefanie Krüger, Florian Battke, Andrea Sprecher, Marita Munz, Matthis Synofzik, Ludger Schöls, Thomas Gasser, Torsten Grehl, Johannes Prudlo, Saskia Biskup

**Affiliations:** ^1^CeGaT GmbH, Center for Genomics and TranscriptomicsTübingen, Germany; ^2^Department of Neurodegenerative Diseases, Hertie-Institute for Clinical Brain Research, University of TübingenTübingen, Germany; ^3^German Research Center for Neurodegenerative DiseasesTübingen, Germany; ^4^Department of Neurology, BG-Kliniken Bergmannsheil GmbH, Ruhr-University BochumBochum, Germany; ^5^Department of Neurology, University of RostockRostock, Germany; ^6^German Research Center for Neurodegenerative DiseasesRostock, Germany

**Keywords:** amyotrophic lateral sclerosis, neurodegeneration, next generation sequencing, genetic heterogeneity, polygenic inheritance

## Abstract

Amyotrophic lateral sclerosis (ALS) is a progressive fatal multisystemic neurodegenerative disorder caused by preferential degeneration of upper and lower motor neurons. To further delineate the genetic architecture of the disease, we used comprehensive panel sequencing in a cohort of 80 German ALS patients. The panel covered 39 confirmed ALS genes and candidate genes, as well as 238 genes associated with other entities of the neurodegenerative disease spectrum. In addition, we performed repeat length analysis for *C9orf72*. Our aim was to (1) identify potentially disease-causing variants, to (2) assess a proposed model of polygenic inheritance in ALS and to (3) connect ALS with other neurodegenerative entities. We identified 79 rare potentially pathogenic variants in 27 ALS associated genes in familial and sporadic cases. Five patients had pathogenic *C9orf72* repeat expansions, a further four patients harbored intermediate length repeat expansions. Our findings demonstrate that a genetic background of the disease can actually be found in a large proportion of seemingly sporadic cases and that it is not limited to putative most frequently affected genes such as *C9orf72* or *SOD1*. Assessing the polygenic nature of ALS, we identified 15 patients carrying at least two rare potentially pathogenic variants in ALS associated genes including pathogenic or intermediate *C9orf72* repeat expansions. Multiple variants might influence severity or duration of disease or could account for intrafamilial phenotypic variability or reduced penetrance. However, we could not observe a correlation with age of onset in this study. We further detected potentially pathogenic variants in other neurodegeneration associated genes in 12 patients, supporting the hypothesis of common pathways in neurodegenerative diseases and linking ALS to other entities of the neurodegenerative spectrum. Most interestingly we found variants in *GBE1* and *SPG7* which might represent differential diagnoses. Based on our findings, we recommend two-staged genetic testing for ALS in Germany in patients with familial and sporadic ALS, comprising *C9orf72* repeat analysis followed by comprehensive panel sequencing including differential diagnoses that impair motor neuron function to meet the complexity of ALS genetics.

## Introduction

Amyotrophic lateral sclerosis (ALS) is a devastating multisystemic neurodegenerative disorder characterized by degeneration of upper and lower motor neurons in the motor cortex, brain stem, and spinal cord (Peters et al., 2015). ALS can be inherited in an autosomal dominant, autosomal recessive or X-linked manner. About 10% of cases are considered as being familial (fALS), whereas the remaining 90% seem to occur sporadically (sALS) with no family history of ALS. Since the first discovery of *SOD1* mutations being causative for ALS1 in 1993 (Rosen et al., 1993), researchers all over the world have made great effort to further delineate the genetic basis underlying ALS. Today, more than 30 confirmed major disease genes are listed by the Amyotrophic Lateral Sclerosis Online genetics Database (ALSoD^1^), the most frequently affected being *C9orf72* (40% fALS, 5–6% sALS; pathogenic repeat expansion in the non-coding region between exons 1a and 1b, detection by repeat analysis), *SOD1* (20% fALS, 3% sALS), *FUS* (5% fALS, <1% sALS) and *TARDBP* (3% fALS, 2% sALS) (Abel et al., 2012; Su et al., 2014).

Screening of the known ALS genes identifies pathogenic mutations in more than 60% of fALS cases. However, the same genes that can be affected in fALS can also be found mutated in sporadic cases, e.g., due to incomplete penetrance, false paternity, recessive inheritance or *de novo* mutations (Su et al., 2014). Mutations in disease genes affect different molecular pathways which promote motor neuron degeneration and include protein misfolding and subsequent aggregation, mitochondrial dysfunction and oxidative stress, impaired RNA processing, glutamate excitotoxicity and impaired axonal transport (Redler and Dokholyan, 2012; Shaw, 2005). These findings provided fundamental insight into basic underlying pathomechanisms and additionally linked ALS to other disease entities like frontotemporal dementia (FTD) or hereditary spastic paraplegia (HSP).

With the application of genome-wide association studies (GWASs) and high throughput sequencing technologies (next generation sequencing, NGS), a large number of additional disease genes, disease modifiers, and risk factors have been identified especially in sALS. GWASs suggest that genetic factors might contribute to a minimum of 23% of disease risk, whereupon such factors do not necessarily have to be directly causative but instead may act as risk factors or disease modifiers (e.g., age of onset, disease progression) in the interplay with environmental and stochastic factors (Renton et al., 2014; Marangi and Traynor, 2015). Numerous GWASs have been published which showed associations of various loci with ALS containing potential risk genes such as *FGGY. ITPR2* and *UNC13A* (Marangi and Traynor, 2015) but until now, causative variants in most of these genes have not been identified. As GWASs are based on the “common disease – common variant” hypothesis and odds ratios associated with risk alleles are usually low, they are solely suitable for the identification of common disease modifiers with low effect size in complex disorders rather than rare causative variants with large effect sizes. By contrast, NGS represents a powerful, groundbreaking approach to detect rare variants with moderate or high penetrance in Mendelian diseases without having access to large pedigrees (He et al., 2014). ALS and other neurodegenerative diseases which are characterized by great genetic heterogeneity and sometimes overlapping symptoms or even atypical phenotypes benefit to a great extent from NGS and the possibility to analyze all genes implicated in the disease in one approach. During the last years, the use of NGS encompassed and considerably increased the number of identified disease genes and risk factors for ALS, generating further insight into underlying pathomechanisms at the same time. One example is the recent discovery of the mitochondrial protein CHCHD10 as being implicated in ALS which for the first time proves a direct impact of mitochondria in the pathogenesis of the disease, a result obtained by exome sequencing in several families affected by ALS (e.g., Bannwarth et al., 2014; Müller et al., 2014; Kurzwelly et al., 2015). As sequencing costs and turnaround times substantially decreased during the last years, the broad application of NGS has triggered a fundamental shift not only in clinical genetics but also in research on rare heritable diseases. Additionally, by the analysis of large numbers of genes in parallel, it has become evident that some patients carry potentially pathogenic variants in genes that are associated with other entities of the neurodegenerative spectrum. Besides this, one emerging theme in ALS genetics is the presumption that ALS might be a complex disease. This view arises mainly from the observation of reduced penetrance in pedigrees affected by fALS and the partially missing heritability in sporadic cases (van Blitterswijk et al., 2012; He et al., 2014). In recent studies, the authors applied NGS to identify patients who carried pathogenic or potentially pathogenic variants in more than one disease gene with frequencies ranging from 1.6% to 31.7% in fALS and sALS cohorts (van Blitterswijk et al., 2012; Kenna et al., 2013; Cady et al., 2015). However, these studies additionally point out that the genetic basis underlying ALS in cohorts of different European countries and the US differs due to founder effects and thus should not be assumed to be homogeneous.

Here we hypothesize that ALS is caused by polygenic contributions from many disease-causing or disease-modifying gene variants which encompass not only known ALS genes but also other genes from the neurodegenerative disease spectrum. To investigate this hypothesis, we used a high-coverage targeted high-throughput sequencing approach to detect variants in 39 ALS associated genes as well as 238 additional genes that are linked to other neurodegenerative diseases in a German cohort of 80 clinically well characterized ALS patients. We aim at identifying known causative mutations and novel variants, to report on patients who carry multiple potentially disease causing variants or variants in genes which are implicated not only in ALS, but also in other neurodegenerative disorders. To our knowledge, this is the first report on extensive genetic screening in a German ALS cohort including not only confirmed ALS genes but also possible candidate genes, modifiers and risk factors to assess the great genetic heterogeneity of ALS in Germany.

## Materials and Methods

### Study Participants

Our cohort includes 80 unrelated clinically diagnosed ALS patients (55% male, 45% female; 7.5% familial, 92.5% sporadic; 82.5% ALS, 6.25% ALS-FTD, 2.5% flail leg, 2.5% flail arm, 6.25% primary lateral sclerosis (PLS)). Mean age of disease onset was 60.1 years (range 29–88 years). Patients were recruited consecutively in ALS outpatient clinics at the university hospitals Rostock and Bochum (Germany). Relationship was excluded by evaluation of family history. Only one affected individual per family was included in this study and there was no evidence of relationship between any study participants. Informed written consent was obtained from all participants. The study was approved by the local medical ethics committee of Rostock University (A2009-10 and A2011-56) and conducted in accordance with the Declaration of Helsinki.

### DNA Extraction

Genomic DNA was extracted from EDTA blood using the QIAamp DNA Blood Mini Kit (Qiagen, Hilden, Germany) according to the manufacturer’s protocol.

### *C9orf72* Repeat Analysis

All subjects were screened for a pathological repeat expansion in the *C9orf72* gene (GenBank NM_018325.3, NM_145005.5) using fragment length analysis of fluorescence labeled PCR products as repeat expansions cannot be detected by NGS (method according to DeJesus-Hernandez et al., 2011). Based on a repeat primed PCR we determined the size of GGGGCC repeats (method according to Renton et al., 2011). Repeat lengths of ≥ 30 units were considered as being pathogenic, whereas repeat lengths of 20 to 29 units are considered as intermediate.

### Targeted Resequencing

Genomic DNA was enriched using a custom design Agilent SureSelect in solution kit. The design of our diagnostic panel for neurodegenerative diseases (277 genes in total) included 14 genes which were classified as disease genes when this study was initiated, 25 putative candidate genes, modifiers, and risk factors identified by literature research as being most presumably implicated in ALS (e.g., by GWAS, experimental evidence, or connected pathways; **Table 1**), as well as 238 genes associated with other neurodegenerative diseases (for example genes associated with FTD, HSP and others; see Supplementary Data, 763 kb in total). Sequencing was performed using barcoded libraries on the SOLiD 5500xl platform according to the manufacturer’s instructions (Fragment Library Preparation 5500 Series SOLiD^TM^ Systems, User Guide, Applied Biosystems by Life Technology). Approximately 2.3 million on target reads were generated per sample and the mean coverage on target was 184.2 sequencing reads with a mean mapping quality of 85.3. On average 89.4% of bases were covered by ≥10 reads/base per sample. The primary data analysis was performed using Lifescope (versions v2.5-r0 and v2.5-r2.5.1).

**Table 1:** Genes analyzed in this study.

Gene	Transcript	Genetic subtype
*SOD1*	NM_000454.4	ALS1
*ALS2*	NM_020919.3	ALS2
*SETX*	NM_015046.5	ALS4
*SPG11*	NM_025137.3	ALS5
*FUS*	NM_004960.3	ALS6
*VAPB*	NM_004738.4	ALS8
*ANG*	NM_001145.4	ALS9
*TARDBP*	NM_007375.3	ALS10
*FIG4*	NM_014845.5	ALS11
*OPTN*	NM_021980.4	ALS12
*ATXN2*	NM_002973.3	ALS13
*VCP*	NM_007126.3	ALS14
*CHMP2B*	NM_014043.3	ALS17
*C9orf72*	NM_018325.3	FTDALS1
*APEX1*	NM_001641.3	
*ATXN1*	NM_000332.2	
*CCS*	NM_005125.1	
*DAO*	NM_001917.4	
*DCTN1*	NM_004082.4	
*DPP6*	NM_001936.4	
*FGGY*	NM_001113411.1	
*GLE1*	NM_001003722.1	
*GRN*	NM_002087.2	
*HEXA*	NM_000520.4	
*HFE*	NM_000410.3	
*ITPR2*	NM_002223.2	
*KIFAP3*	NM_014970.3	
*LIF*	NM_002309.4	
*NAIP*	NM_004536.2	
*NEFH*	NM_021076.3	
*PON1*	NM_000446.5	
*PON2*	NM_000305.2	
*PON3*	NM_000940.2	
*RNF19A*	NM_183419.3	
*SLC1A2*	NM_004171.3	
*SPAST*	NM_014946.3	
*UNC13A*	NM_001080421.2	
*VEGFA*	NM_001025366.2	
*VPS54*	NM_001005739.1	


*The top 14 genes were classified as disease genes when this study was initiated; a further 25 candidate genes, modifiers and risk factors were also included. Gene names are HGNC symbols, transcripts are identified by RefSeq accessions.*

### Variant Filtering

Only variants (SNVs/small indels) with a minor allele frequency (MAF) of ≤1% in coding and flanking intronic regions (±8 base pairs) and the UTR regions were evaluated. Known disease causing mutations which are listed in the HGMD database were evaluated in coding and flanking intronic regions up to ±30 base pairs and up to a MAF of ≤5%. Population frequencies are adapted from the following databases: 1000 Genomes, dbSNP, Exome Variant Server, ExAC and an internal database. Our quality criteria required coverage of ≥10 quality reads per base and a novel allele frequency (NAF) of ≥0.3. Detected variants were assessed based on their MAF, current literature and widely used Online databases [e.g., OMIM (McKusick-Nathans Institute of Genetic Medicine, Johns Hopkins University, Baltimore, MD, USA), HGMD (Stenson et al., 2014), Uniprot (UniProt Consortium, 2015), locus or disease specific databases] and prediction tools [MutationTaster (Schwarz et al., 2014), PolyPhen2 (Adzhubei et al., 2010), SIFT (Choi et al., 2012), NetGene2 Server (Brunak et al., 1991) and Splice Site Prediction by Neural Network (Reese et al., 1997)].

### Comparison of Observed Frequencies

We compared the observed frequencies of affected genes in ALS cohorts from the US (Couthouis et al., 2014), Ireland (Kenna et al., 2013), Italy (Chiò et al., 2012) and Great Britain (Morgan et al., 2015) with detected frequencies in our cohort.

### Generation of a Protein–Protein Interaction Network

To visually link candidate genes and possible modifiers to ALS, and to put them in relation to each another and to confirmed ALS genes, we created a protein–protein interaction network containing 21 disease genes and 13 candidate genes, possible risk factors, and modifiers covered by our sequencing panel (**Figure 2**). The protein-protein interaction network was created using the STRING database v10^2^ by searching for multiple proteins: ALS2, ANG, ATXN1, ATXN2, C9orf72, CHCHD10, CHMP2B, DPP6, ERBB4, FGGY, FIG4, FUS, GBE1, GLE1, GRN, HNRNPA1, ITPR2, KIFAP3, MATR3, NEFH, OPTN, PFN1, PON3, SETX, SIGMAR1, SLC2A1, SOD1, SPG11, SPG7, TARDBP, UBQLN2, UNC13A, VAPB, VCP. Standard settings were used, network edges set to show confidence, and structural previews inside network bubbles were disabled.

## Results

### Identification of Variants in ALS Associated Genes

**FIGURE 1:**
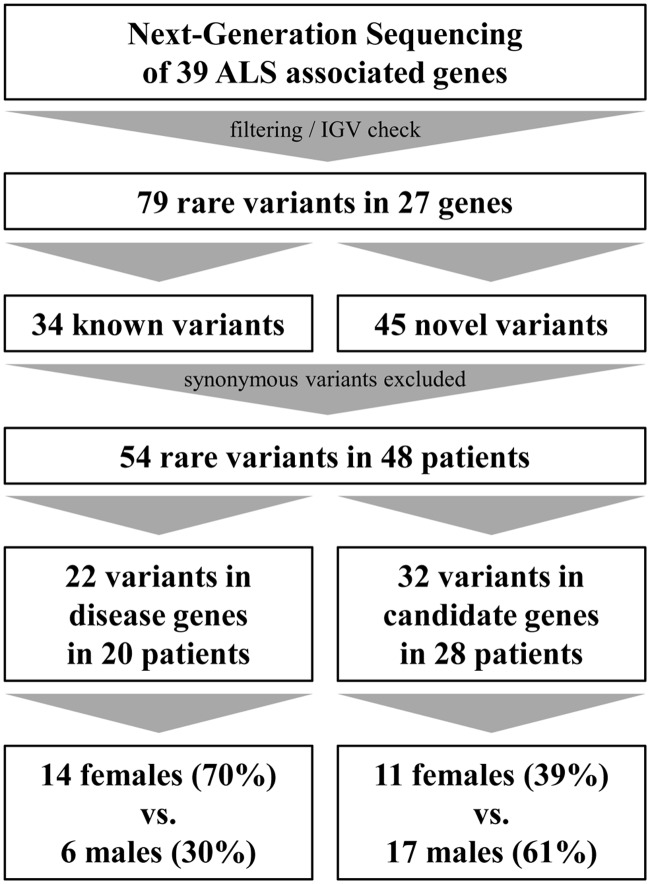
**Detection and filtering of variants**.

By analyzing 39 ALS associated genes (**Table 1**), we were able to detect 79 rare variants (European–American MAF ≤ 1% in dbSNP, EVS or ExAC) in 27 genes which passed defined filter criteria (see Variant Filtering) and manual assessment in the Integrated Genome Viewer (IGV, v2.1.28 rev release 175, Robinson et al., 2011; see **Table 2**). Of these, 34 variants have been published previously whereas 45 have not been described before and therefore are considered as being novel. Excluding synonymous substitutions, we identified 54 rare variants in 23 male and 25 female patients (48 patients representing 60% of our cohort). We found that 20 patients of whom 95% (19 out of 20 patients) are considered as sporadic cases carry variants in 14 known disease genes. Additionally we identified variants in candidate genes, modifiers or risk factors in 28 patients (see **Figure 1**).

**Table 2:** Identified variants in ALS associated genes.

Gene	cDNA	Protein	Zygosity	MAF_EA (%)	MAF (%) in this study	dbSNP	Pat-ID	Gender	Subtype	AAO (years)	Reference	MT	PolyPhen2	SIFT	NG2	NN
**ALS disease genes**											

*ALS2*	c.4119A > G	p.I1373M	het	0.52	0.63	rs61757691	#422	f	sALS	61	Kenna et al., 2013	Disease causing	benign	tolerated	–	–
*ALS2*	c.1816-8C > T	p.?	het	0.25	1.25	rs185911369	#26^∗∗^	m	PLS	66	–	Polymorphism	–	–	no effect	no effect
*ALS2*	c.1816-8C > T	p.?	het	0.25	1.25	rs185911369	#524	f	sALS	60	–	Polymorphism	–	–	no effect	no effect
*ALS2*	c.1127_1129 delAAG	p.E375del	het	–	0.63	–	#45	f	sPLS	43	–	Disease causing	–	–	–	–
*ATXN2*	c.2049A > T	p.L683F	het	–	0.63	–	#34	f	ALS-FTD	54	–	Disease causing	possibly damaging	damaging	–	–
*C9orf72*	c.956C > A	p.P319Q	het	–	0.63	–	#37a	m	sALS	50	–	Disease causing	probably damaging	tolerated	–	–
*FIG4*	c.1940A > G	p.Y647C	het	0.01	0.63	rs150301327	#10^∗^	m	sALS	71	Chow et al., 2009	Disease causing	benign	damaging	–	–
*FIG4*	c.1910C > A	p.P637Q	het	–	0.63	–	#44	f	ALS	88	–	Polymorphism	benign	tolerated	–	–
*SETX*	c.3229G > A	p.D1077N	het	0.14	0.63	rs145097270	#571	m	sALS	49	Kenna et al., 2013	Polymorphism	possibly damaging	damaging	–	–
*SETX*	c.7358A > G	p.K2453R	het	–	0.63	–	#29	f	sALS	73	–	Polymorphism	benign	tolerated	–	–
*SPG11*	c.6950G > A	p.G2317D	het	<0.01	0.63	rs79186522	#23	f	flail leg	69	–	Polymorphism	benign	tolerated	–	–
*SPG11*	c.5381T > C	p.L1794P	het	<0.01	0.63	rs201689565	#729^∗∗^	m	sALS	40	–	Disease causing	probably damaging	damaging	–	–
*SPG11*	c.3577A > G	p.I1193V	het	0	0.63	–	#747	f	sALS	69	–	Polymorphism	benign	tolerated	–	–
*TARDBP*	c.931A > G	p.M311V	het	–	0.63	rs80356725	#741	f	sALS	64	Lemmens et al., 2009	Disease causing	benign	tolerated	–	–
*VAPB*	c.166C > T	p.P56S	het	–	0.63	rs74315431	#3	m	fALS	41	Nishimura et al., 2004; Aliaga et al., 2013	Disease causing	probably damaging	damaging	–	–
*VAPB*	c.390T > G	p.D130E	het	0.15	0.63	rs146459055	#22	f	sALS	67	Suzuki et al., 2009	Disease causing	benign	tolerated	–	–
*VAPB*	c.479_481 delCTT	p.S160del	het	0.28	0.63	rs566283411	#677	f	sALS	72	Landers et al., 2008	Disease causing	–	–	–	–
*VCP*	c.1194+3G > A	p.?	het	0.05	0.63	rs183223259	#20	f	ALS-FTD	70	–	Disease causing	–	–	no effect	effect

**ALS candidate genes, modifiers, risk factors**											

*ATXN1*	c.1117C > T	p.R373C	het	<0.01	0.63	–	#34	f	ALS-FTD	54	–	Disease causing	Probably damaging	Damaging	–	–
*ATXN1*	c.511C > A	p.R171S	het	0	0.63	–	#38	f	ALS-FTD	70	–	Disease causing	Probably damaging	Damaging	–	–
*DPP6*	c.746C > T	p.T249M	het	–	0.63	–	#428	m	sALS	64	–	Disease causing	Possibly damaging	Tolerated	–	–
*FGGY*	c.1221 + 2T > C	p.?	het	0.45	3.13	rs41287704	#5	m	sALS	58	Kenna et al., 2013	Disease causing	–	–	Effect	Effect
*FGGY*	c.1221 + 2T > C	p.?	het	0.45	3.13	rs41287704	#21	f	sALS	61	Kenna et al., 2013	Disease causing	–	–	Effect	Effect
*FGGY*	c.1221 + 2T > C	p.?	het	0.45	3.13	rs41287704	#732	m	sALS	61	Kenna et al., 2013	Disease causing	–	–	Effect	Effect
*FGGY*	c.1221 + 2T > C	p.?	het	0.45	3.13	rs41287704	#739	m	sALS	72	Kenna et al., 2013	Disease causing	–	–	Effect	Effect
*FGGY*	c.1221 + 2T > C	p.?	het	0.45	3.13	rs41287704	#33	m	sALS	63	Kenna et al., 2013	Disease causing	–	–	Effect	Effect
*FGGY*	c.1435T > C	p.C479R	het	<0.01	0.63	–	#703	m	fALS	76	–	Disease causing	Probably damaging	Damaging	–	–
*FGGY*	c.979A > C	p.N327H	het	0.13	1.25	rs34026954	#28	f	ALS	67	Kenna et al., 2013	Disease causing	Probably damaging	Damaging	–	–
*FGGY*	c.979A > C	p.N327H	het	0.13	1.25	rs34026954	#38	f	ALS-FTD	70	Kenna et al., 2013	Disease causing	Probably damaging	Damaging	–	–
*GLE1*	c.398G > A	p.R133Q	het	<0.01	0.63	–	#37b	m	sALS	74	–	Disease causing	Benign	Tolerated	–	–
*GRN*	c.545C > T	p.T182M	het	0.03	0.63	rs63750479	#16	m	sALS	67	Guerreiro et al., 2010	Polymorphism	Benign	Tolerated	–	–
*GRN*	c.229G > A	p.V77I	het	0.01	0.63	rs148531161	#749	m	sALS	46	Yu et al., 2010	Polymorphism	Benign	Tolerated	–	–
*GRN*	c.361G > A	p.V121M	het	0	0.63	–	#28	f	ALS	67	–	Polymorphism	Benign	Damaging	–	–
*GRN*	c.970G > A	p.A324T	het	0.12	0.63	rs63750541	#36	m	flail arm	39	Sleegers et al., 2008; Kenna et al., 2013	Polymorphism	Benign	Tolerated	–	–
*ITPR2*	c.2831C > T	p.P944L	het	<0.01	0.63	rs377598368	#22	f	sALS	67	–	Disease causing	Benign	Tolerated	–	–
*ITPR2*	c.3485T > G	p.V1162G	het	0.15	0.63	rs61757114	#373^∗^	f	sALS	72	Kenna et al., 2013	Disease causing	Benign	Tolerated	–	–
*ITPR2*	c.1834G > A	p.A612T	het	<0.01	0.63	rs199523133	#422	f	sALS	61	–	Disease causing	Possibly damaging	Tolerated	–	–
*ITPR2*	c.8002G > A	p.A2668T	het	0.21	0.63	rs61757116	#677	f	sALS	72	Kenna et al., 2013	Disease causing	Benign	Tolerated	–	–
*ITPR2*	c.3635C > T	p.A1212V	het	<0.01	0.63	rs368911384	#741	f	sALS	64	Kenna et al., 2013	Disease causing	Probably damaging	Tolerated	–	–
*ITPR2*	c.1447G > A	p.V483I	het	0	0.63	–	#29	f	sALS	73	–	Disease causing	Probably damaging	Tolerated	–	–
*ITPR2*	c.3539G > A	p.R1180Q	het	0.62	0.63	rs35862420	#36	m	flail arm	39	Kenna et al., 2013	Disease causing	Benign	Tolerated	–	–
*KIFAP3*	c.518-5T > A	p.?	het	–	0.63	–	#419	m	sALS	72	–	Polymorphism	–	–	Effect	No effect
*KIFAP3*	c.1301T > G	p.F434C	het	0.23	0.63	rs116755924	#52	m	sALS	45	–	Disease causing	Probably damaging	Damaging	–	–
*NEFH*	c.1235G > A	p.R412Q	het	0.01	0.63	–	#534	m	sALS	58	–	Disease causing	Possibly damaging	Damaging	–	–
*PON3*	c.217G > T	p.G73C	het	–	0.63	–	#47	m	sALS	78	–	Disease causing	Probably damaging	Damaging	–	–
*SLC1A2*	c.236C > G	p.A79G	het	0.04	0.63	rs377633002	#524	f	sALS	60	Meyer et al., 1998	Disease causing	Benign	Tolerated	–	–
*UNC13A*	c.3080C > T	p.P1027L	het	1.83	0.63	rs200328448	#10^∗^	m	sALS	71	Koppers et al., 2013; Kenna et al., 2013	Disease causing	Possibly damaging	Damaging	–	–
*UNC13A*	c.182C > T	p.T61M	het	0.45	0.63	rs140141294	#26^∗∗^	m	PLS	66	Koppers et al., 2013; Kenna et al., 2013	Disease causing	Possibly damaging	Tolerated	–	–
*UNC13A*	c.1073A > G	p.Y358C	het	–	0.63	–	#30	m	sALS	60	–	Polymorphism	Possibly damaging	Tolerated	–	–

**UTR variants**						

*APEX1*	c.^∗^2A > T	p.?	het	0.5	0.63	rs17112002	#47	m	sALS	78	–					
*FUS*	c.-37C > T	p.?	het	–	0.63	–	#422	f	sALS	61	–					
*FUS*	c.^∗^41G > A	p.?	het	0.86	0.63	rs80301724	#741	f	sALS	64	Sproviero et al., 2012					
*SOD1*	c.-8A > C	p.?	het	–	0.63	–	#46	f	ALS-FTD	75	–					
*VAPB*	c.-33C > G	p.?	het	–	0.63	rs201547974	#676	f	sALS	51	–					

**Synonymous variants**						

*ATXN2*	c.2088C > T	p.(=)	het	–	0.63	–	#22	f	sALS	67	–					
*DAO*	c.723C > T	p.(=)	het	0.23	1.25	rs149956241	#25^∗∗^	f	flail leg	54	–					
*DAO*	c.723C > T	p.(=)	het	0.23	1.25	rs149956241	#41	m	sALS	42	–					
*DCTN1*	c.3669T > C	p.(=)	het	0	0.63	–	#12	m	sALS	61	–					
*DCTN1*	c.3474A > G	p.(=)	het	–	0.63	–	#54	f	ALS-FTD	55	–					
*DPP6*	c.693T > C	p.(=)	het	–	0.63	–	#24^∗^	m	sALS	70	Kenna et al., 2013					
*FUS*	c.1080C > T	p.(=)	het	0.05	0.63	rs190724342	#35	f	sALS	49	Kenna et al., 2013					
*HEXA*	c.1216C > T	p.(=)	het	0.02	0.63	rs140482769	#7	m	sALS	71	–					
*HEXA*	c.744C > T	p.(=)	het	–	0.63	–	#749	m	sALS	46	–					
*ITPR2*	c.4962G > A	p.(=)	het	0.69	0.63	rs191789657	#16	m	sALS	67	Kenna et al., 2013					
*ITPR2*	c.5569C > T	p.(=)	het	0.12	1.25	rs191281974	#24^∗^	m	sALS	70	Kenna et al., 2013					
*ITPR2*	c.5569C > T	p.(=)	het	0.12	1.25	rs191281974	#40	f	sALS	43	Kenna et al., 2013					
*ITPR2*	c.6162C > T	p.(=)	het	<0.01	0.63	–	#31	f	sALS	47	–					
*NEFH*	c.2061A > G	p.(=)	het	–	0.63	–	#16	m	sALS	67	–					
*NEFH*	c.2646C > T	p.(=)	het	0.01	0.63	rs528790943	#422	f	sALS	61	Kenna et al., 2013					
*PON1*	c.603G > A	p.(=)	het	0.17	0.63	rs148452713	#729^∗∗^	m	sALS	40	Kenna et al., 2013					
*SETX*	c.6675C > T	p.(=)	het	<0.01	0.63	rs200382898	#33	m	sALS	63	-					
*SLC1A2*	c.846C > A	p.(=)	het	<0.01	0.63	rs376593061	#46	f	ALS-FTD	75	-					
*SLC1A2*	c.450G > A	p.(=)	het	0	0.63	-	#52	m	sALS	45	-					
*SPG11*	c.6258G > T	p.(=)	het	0.81	0.63	rs150761878	#13	m	sALS	73	Kenna et al., 2013					
*UNC13A*	c.771C > G	p.(=)	het	3.02	0.63	rs146739681	#3	m	fALS	41	Kenna et al., 2013					
*UNC13A*	c.4560C > T	p.(=)	het	0.1	0.63	rs141334897	#26^∗∗^	m	PLS	66	-					
*UNC13A*	c.4143G > A	p.(=)	het	-	0.63	-	#26^∗∗^	m	PLS	66	-					
*UNC13A*	c.2220G > A	p.(=)	het	0.17	0.63	rs201361019	#32	m	sALS	46	-					
*VCP*	c.832T > C	p.(=)	het	0.04	0.63	rs200670526	#625	m	fALS	53	-					


*^∗^Patients carry ≥30 *C9orf72* repeat units. ^∗∗^Patients carry intermediate lenght *C9orf72* repeats. MAF, minor allele frequency; MAF_EA is the maximum population frequency of the variant observed in the European American population in dbSNP, EVS or ExAC. AAO, age at onset; MT, MutationTaster (http://www.mutationtaster.org/); PolyPhen2 (http://genetics.bwh.harvard.edu/pph2/); SIFT (http://provean.jcvi.org/genome_submit_2.php); NG2, NetGene2 (https://www.cbs.dtu.dk/services/NetGene2/); NN, Splice Site Prediction by Neural Network (http://www.fruitfly.org/seq_tools/splice.html).*

Pathogenic repeat expansions in the *C9orf72* gene were identified in five (6.25%) sporadic patients (mean age of onset: 67.6 years, range 49–76 years). Two of these patients carried additional variants in *FIG4* and *UNC13A* (pat #10), and *ITPR2* (pat #373), respectively (see **Table 3**). Furthermore, we identified four patients carrying intermediate length repeat expansions (mean age of onset: 57 years, range 40–68 years). Of these, two individuals carried additional missense and splice variants in *ALS2* and *UNC13A* (pat #26), and *SPG11* (pat #729) respectively (see **Table 2**). Given the size of this sample, the remarkable difference in mean age of onset between the patients with intermediate length expansions and carriers of pathogenic repeat expansions is not statistically significant (*p* = 0.11, Wilcoxon–Mann–Whitney test).

**Table 3:** Carriers of pathogenic and intermediate *C9orf72* repeat expansions.

Pat-ID	Gender	Subtype	AAO (years)	*C9orf72*	n repeats	Additional variants
#2	m	sALS	49	Positive	30	
#10	m	sALS	71	Positive	35	*FIG4* c.1940A > G; p.Y647C (het.); *UNC13A* c.3080C > T; p.P1027L (het.)
#24	m	sALS	70	Positive	31	
#373	f	sALS	72	Positive	34	*ITPR2* c.3485T > G; p.V1162G (het.)
#673	f	sALS	76	Positive	n.a.	
#6	m	sALS	68	Intermediate	26	
#25	f	flail leg syndrome	54	Intermediate	27	
#26	m	sPLS	66	Intermediate	27	*UNC13A* c.182C > T; p.T61M (het.); *ALS2* c.1816-8C > T; p.?
#729	m	sALS	40	Intermediate	27	*SPG11* c.5381T > C; p.L1794P (het.)


By focusing on candidate genes, modifiers, and risk factors, one interesting finding is the identification of four missense variants in the *GRN* gene (see **Table 2**). Of these variants, three have already been described as being probably benign in FTD cases (p.T182M), of unknown clinical relevance in FTD and progressive non-fluent aphasia (p.A324T), or as being potentially pathogenic in FTD spectrum disease (p.V77I), respectively (Guerreiro et al., 2008; Pickering-Brown et al., 2008; Yu et al., 2010). Besides this, we detected seven missense variants in the *ITPR2* gene which was linked to ALS by several GWASs in the past (van Es et al., 2007), eight variants in *FGGY*, and three variants in *UNC13A*, as well as variants in *ATXN1. DPP6. GLE1. KIFAP3. NEFH. PON3* and *SLC1A2* (see **Table 2**).

### Co-occurrence of Variants in ALS Associated Genes

**FIGURE 2:**
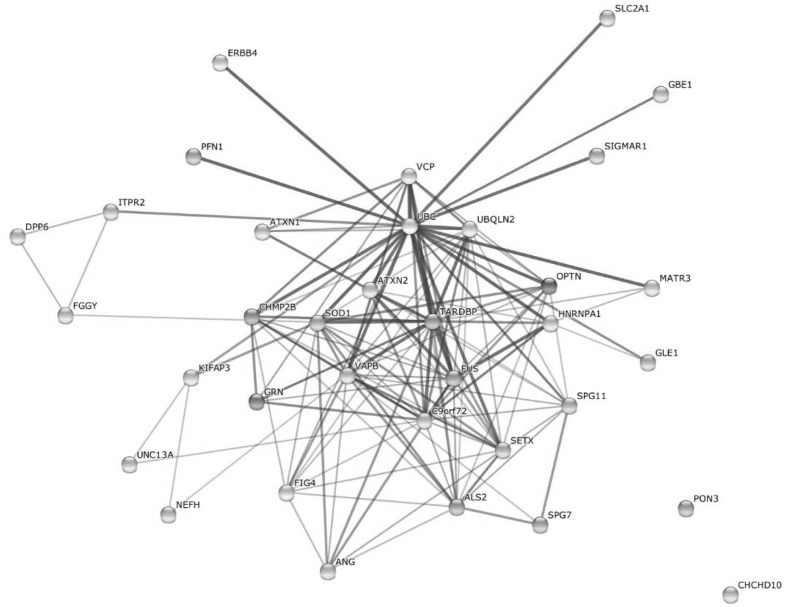
**Protein–protein-interaction network including 21 known ALS genes as well as 13 candidate genes, risk factors and possible modifiers included in our ALS gene panel (created with STRING10, Jensen et al., 2009).** UBC, Ubiquitin C.

Earlier studies supported a complex genetic basis for ALS, which is also supported by protein–protein interactions between known ALS-associated genes, candidate genes, risk factors, and possible modifiers included in our gene panel (**Figure 2**). In our example, each of the proteins interacts in the context of key proteins for motor neuron degeneration (except *CHCHD10* and *PON3*), pointing toward possible modifying effects of certain variants.

We searched our cohort for patients who carry multiple variants in ALS-associated genes and could identify 15 individuals carrying at least two variants (18.8%, synonymous variants excluded) in ALS-associated genes (**Table 4**). For example, missense variants in the *ITPR2* gene were found in co-occurrence with clearly or potentially pathogenic variants in seven (8.75%) individuals. Four of these variants were also detected in an ALS cohort screening by Kenna et al. (2013). In our cohort the mean age of onset in patients who carried a variant in the *ITPR2* gene in co-occurrence was 64.0 years compared to 66.6 years in patients carrying any other variants in co-occurrence (differences are not statistically significant, Wilcoxon–Mann–Whitney test). We detected two additional synonymous variants in *ITPR2* but according to current knowledge we cannot assess their actual impact on the ITPR2 protein. Four of the 15 patients carried an expanded (Pat #10 and Pat #373) or intermediate (Pat #26 and Pat #729) *C9orf72* repeat expansion in co-occurrence.

**Table 4:** Co-occurrence of variants in ALS associated genes.

Pat-ID	Gender	Subtype	AAO (years)	Gene	cDNA	Protein	MAF_EA (%)	dbSNP
#10	m	sALS	71	*C9orf72*	Pathogenic repeat expansion			
					
				*FIG4*	c.1940A > G	p.Y647C	0.02	rs150301327


				*UNC13A*	c.3080C > T	p.P1027L	0.65	rs200328448
#22	f	sALS	67	*ATXN2*	c.2088C > T	p.(=)	-	-
				*ITPR2*	c.2831C > T	p.P944L	0.01	rs377598368
				*VAPB*	c.390T > G	p.D130E	0.1	rs146459055
					
#26	m	PLS	66	*C9orf72*	Intermediate repeat expansion			
					
				*ALS2*	c.1816-8C > T	p.?	0.38	rs185911369
				*UNC13A*	c.4560C > T	p.(=)	0.07	rs141334897
				*UNC13A*	c.4143G > A	p.(=)	-	-
				*UNC13A*	c.182C > T	p.T61M	0.2	rs140141294
#422	f	sALS	61	*ALS2*	c.4119A > G	p.I1373M	0.49	rs61757691
				*FUS*	c.-37C > T	p.?	-	-
				*ITPR2*	c.1834G > A	p.A612T	0.01	rs199523133
				*NEFH*	c.2646C > T	p.(=)	-	-
#524	f	sALS	60	*ALS2*	c.1816-8C > T	p.?	0.38	rs185911369
				*SLC1A2*	c.236C > G	p.A79G	0.01	rs377633002
#677	f	sALS	72	*ITPR2*	c.8002G > A	p.A2668T	0.29	rs61757116
				*VAPB*	c.479_481delCTT	p.S160del	0.45	rs566283411
#741	f	sALS	64	*FUS*	c.^∗^41G > A	p.?	0.58	rs80301724
				*ITPR2*	c.3635C > T	p.A1212V	-	rs368911384
				*TARDBP*	c.931A > G	p.M311V	-	rs80356725
#29	f	sALS	73	*ITPR2*	c.1447G > A	p.V483I	-	-
				*SETX*	c.7358A > G	p.K2453R	-	-
#28	f	ALS	67	*FGGY*	c.979A > C	p.N327H	0.1	rs34026954
				*GRN*	c.361G > A	p.V121M	-	-
#34	f	ALS-FTD	54	*ATXN1*	c.1117C > T	p.R373C	-	-
				*ATXN2*	c.2049A > T	p.L683F	-	-
#36	m	flail arm	39	*GRN*	c.970G > A	p.A324T	0.14	rs63750541
				*ITPR2*	c.3539G > A	p.R1180Q	0.76	rs35862420
#38	f	ALS-FTD	70	*ATXN1*	c.511C > A	p.R171S	-	-
				*FGGY*	c.979A > C	p.N327H	0.1	rs34026954
#47	m	sALS	78	*APEX1*	c.^∗^2A > T	p.?	0.66	rs17112002
				*PON3*	c.217G > T	p.G73C	-	-
					
#373	f	sALS	72	*C9orf72*	Pathogenic repeat expansion			
					
				*ITPR2*	c.3485T > G	p.V1162G	0.15	rs61757114
					
#729	m	sALS	40	*C9orf72*	Intermediate repeat expansion			
					
				*PON1*	c.603G > A	p.(=)	0.12	rs148452713
				*SPG11*	c.5381T > C	p.L1794P	0.01	rs201689565
#3	m	fALS	41	*UNC13A*	c.771C > G	p.(=)	0.85	rs146739681
				*VAPB*	c.166C > T	p.P56S	-	rs74315431
#16	m	sALS	67	*GRN*	c.545C > T	p.T182M	0.03	rs63750479
				*ITPR2*	c.4962G > A	p.(=)	0.36	rs191789657
				*NEFH*	c.2061A > G	p.(=)	-	-
					
#24	m	sALS	70	*C9orf72*	pathogenic repeat expansion			
					
				*DPP6*	c.693T > C	p.(=)	-	-
				*ITPR2*	c.5569C > T	p.(=)	0.17	rs191281974
#749	m	sALS	46	*GRN*	c.229G > A	p.V77I	0.01	rs148531161
				*HEXA*	c.744C > T	p.(=)	-	-
#33	m	sALS	63	*FGGY*	c.1221+2T > C	p.?	0.45	rs41287704
				*SETX*	c.6675C > T	p.(=)	0.01	rs200382898
#46	f	ALS-FTD	75	*SLC1A2*	c.846C > A	p.(=)	-	-
				*SOD1*	c.-8A > C	p.?	-	-
#52	m	sALS	45	*KIFAP3*	c.1301T > G	p.F434C	0.23	rs116755924
				*SLC1A2*	c.450G > A	p.(=)	-	-


The mean age of onset in patients where no variant could be detected was 57.8 years, patients who carried one variant showed a mean age of onset of 61.3 years and patients carrying two or more variants had a mean age of disease onset of 65.0 years. In comparison, the overall mean age of disease onset in our cohort was 60.1 years. However, these differences in age of onset are not statistically significant (Kruskal–Wallis Rank Sum Test).

### Variants in Other NDD Genes

To match the hypothesis of common pathways in different neurodegenerative diseases (NDDs) and to link ALS to other entities of the NDD spectrum, we additionally searched for potentially pathogenic or disease causing variants in 238 genes which are associated with possible differential diagnoses or overlapping phenotypes that are included in our NDD gene panel. We identified 12 patients who carried potentially pathogenic variants in genes that are linked to other entities (**Table 5**).

**Table 5:** Detected variants in other NDD genes.

Pat-ID	Gender	Subtype	AAO (years)	Gene	chr. position	cDNA	Protein	Zygosity	MAF_EA (%)	dbSNP	Differential diagnoses (OMIM)	MT	PolyPhen2	SIFT	NG2	NN
#5	m	sALS	58	*AR*	chrX:66941751	c.2395C > G	p.Q799E	hemi	0.22	rs137852591	#300068, #312300, #300633, #313200	Disease causing	-	Damaging	-	-
#9	m	sALS	66	*GBA*	chr1:155209737	c.247C > T	p.R83C	het	0.01	rs1141812	#608013, #230800, #230900, #231000, #231005, #127750, #168600	Polymorphism	Probably damaging	Damaging	-	-
#18	m	sALS	42	*PLP1*	chrX:103043442	c.696+3G > A	p.?	hemi	-	-	#312920, #312080	Disease causing	-	-	No effect	No effect
#24^∗^	m	sALS	70	*GARS*	chr7:30665930	c.1694T > A	p.L565Q	het	0.02	rs200726600	#601472, #600794	Disease causing	Benign	Damaging	-	-
#385	f	sALS	69	*GARS*	chr7:30655580	c.1100A > G	p.N367S	het	0.04	rs192443850		Disease causing	Benign	Tolerated	-	-
				*SPG7*	chr16:89592755	c.637C > T	p.R213^∗^	het	-	-	#607259	Disease causing	-	-	-	-
#780	m	fALS	56	*DYNC1H1*	chr14:102452918	c.2356C > T	p.R786C	het	-	-	#614228, #614563, #158600	Disease causing	Probably damaging	Damaging	-	-
#34	f	ALS-FTD	54	*DYNC1H1*	chr14:102508609	c.12259G > A	p.A4087T	het	-	-		Disease causing	Probably damaging	Damaging	-	-
#38	f	ALS-FTD	70	*GBE1*	chr3:81640290	c.1134T > G	p.S378R	het	0.04	rs36099971	#263570, #232500	Disease causing	Benign	Tolerated	-	-
				*GBE1*	chr3:81810551	c.118C > A	p.P40T	het	0.17	rs35196441		Disease causing	Benign	Damaging	-	-
#41	m	sALS	42	*SPG7*	chr16:89623341	c.2228T > C	p.I743T	het	-	-	#607259	Disease causing	Probably damaging	Damaging	-	-
#19	m	sALS	54	*SPG7*	chr16:89598369	c.1045G > A	p.G349S	het	0.17	rs141659620		Disease causing	Probably damaging	Damaging	-	-
				*SYNE1*	chr6:152712567	c.7870C > T	p.R2624W	het	-	-	#612998, #610743	Polymorphism	Probably damaging	Damaging	-	-
#28	f	sALS	67	*SPG7*	chr16:89598369	c.1045G > A	p.G349S	het	0.17	rs141659620	#607259	Disease causing	Probably damaging	Damaging	-	-
#32	m	sALS	46	*TAF1*	chrX:70680560	c.5366A > G	p.N1789S	hemi	0.03	rs147517498	#314250	Disease causing	Benign	Damaging	-	-


*Patient carries a pathogenic *C9orf72* repeat expansion.

In patient #38, we detected two heterozygous variants in the *GBE1* gene (p.S378R and p.P40T, see **Table 5**). Mutations in *GBE1* can cause autosomal recessively inherited adult Polyglucosan body disease (APBD) which is characterized by upper motor neuron signs similar to ALS, early neurogenic bladder, cognitive impairment and decreased or absent activity of the glycogen branching enzyme (Klein, 2013). APBD is one of the conditions that should be considered when establishing the diagnosis of ALS. Unfortunately, we could not investigate whether both variants occur in the compound-heterozygous state in our patient because samples for segregation analysis could not be obtained. Long-range PCR with mutation-specific primers was impossible due to the large distance of more then 170 kb between the variants.

Another interesting finding is the identification of heterozygous variants in the *SPG7* gene in four sporadic patients (see **Table 5**). Mutations in *SPG7* can cause autosomal recessively inherited spastic paraplegia type 7, but there are also some published cases of obviously autosomal dominant inheritance (e.g., Sánchez-Ferrero et al., 2013). The disease is mainly characterized by spasticity and weakness of the lower limbs. Additional neurologic symptoms might appear in more complex phenotypes. In our cohort, we identified the truncating mutation p.R213* and the missense mutations p.I743T and p.G349S which are both described as acting disadvantageous on SPG7 protein function (Brugman et al., 2008; Bonn et al., 2010). None of the four patients had further relevant variants in ALS associated genes (only one patient carries an additional missense variant of unknown clinical relevance in the *FGGY* gene).

We also identified a high number of variants in the *NOTCH3. SYNE1*, and *VPS13A* genes as expected in genes of this size. For *SYNE1*, as mainly loss-of-function mutations are considered as being pathogenic in motor neuron disease (Gros-Louis et al., 2007; Izumi et al., 2013; Noreau et al., 2013). Similarly, only variants which result in a loss or gain of one cysteine residue within epidermal growth factor (EGF)-like repeat domains (Dichgans et al., 2001) are considered pathogenic in *NOTCH3*, and for *VPS13A* mostly loss-of-function variants are considered as pathogenic (Tomiyasu et al., 2011). Thus we assume that detected variants in our cohort represent rare polymorphisms. We identified variants in further genes that are included in our gene panel (see **Table 5**) but are unlikely to be implicated in our patients’ phenotypes.

By comparing the number of patients identified to carry potentially pathogenic variants in ALS related genes in our cohort with previously published cohort studies, we show that the frequency of affected genes may vary in different populations (**Table 6**). For example, in the *VAPB* gene we detected variants in 5% of German patients (four cases) whereas in other populations no variants in *VAPB* were identified at all. Striking differences in frequencies across populations can also be observed for *FIG4. FGGY. GRN. ITPR2*, and *UNC13A*. The studies used vastly different strategies for sequencing and variant evaluation and analyzed different gene sets [from 6 genes, partially hotspots only sequenced by Sanger in Chiò et al. (2012) to 169 genes sequenced by NGS in Couthouis et al. (2014)]. Thus we consider this comparison solely to hint toward possible differences in gene frequencies among populations as a consequence of founder effects.

**Table 6:** Percentage of patients carrying potentially pathogenic variants in ALS associated genes (missense, splicing, small Indels only) (American: Couthouis et al., 2014; Irish: Kenna et al., 2013; Italian: Chiò et al., 2012; British: Morgan et al., 2015).

Gene	Our cohort (%) *n* = 80	American (%) *n* = 242	Irish (%) *n* = 444	Italian (%) *n* = 475	British (%) *n* = 95
*SOD1*	1.25	1.65	0	2.1	2.11
*ALS2*	5	1.24	1.35	-	5.26
*SETX*	2.5	2.07	2.25	–	–
*SPG11*	3.75	4.13	1.58	–	17.89
*FUS*	2.5	0.41	0.45	0.21	1.05
*VAPB*	5	0	0	–	–
*ANG*	0	0.41	0	0	–
*TARDBP*	1.25	–	0.45	1.47	2.11
*FIG4*	2.5	0.83	0	–	–
*OPTN*	0	0	0.23	0.21	2.11
*ATXN2*	1.25	1.22	0	–	–
*VCP*	1.25	0	0.23	–	–
*CHMP2B*	0	0	0.45	–	–
*C9orf72*-Repeat	6.25	1.65	8.78	6.74	–
*APEX1*	1.25	–	–	–	–
*ATXN1*	2.5	–	–	–	–
*CCS*	0	–	–	–	–
*DAO*	0	–	–	–	–
*DCTN1*	0	2.07	0.45	–	–
*DPP6*	1.25	1.65	0.23	–	–
*FGGY*	10	0.41	0.23	–	–
*GLE1*	1.25	–	–	–	–
*GRN*	5	0.41	0	–	–
*HEXA*	0	–	–	–	–
*HFE*	0	1.65	0.23	–	–
*ITPR2*	8.75	1.24	0.23	–	–
*KIFAP3*	2.5	–	–	–	–
*LIF*	0	–	–	–	–
*NAIP*	0	–	–	–	–
*NEFH*	1.25	0.41	0	–	–
*PON1*	0	0	0	–	1.05
*PON2*	0	0	0.23	–	1.05
*PON3*	1.25	0	0	–	–
*RNF19A*	0	–	–	–	–
*SLC1A2*	1.25	–	–	–	–
*SPAST*	0	–	–	–	–
*UNC13A*	3.75	1.24	0.23	–	–
*VEGFA*	0	–	–	–	1.05
*VPS54*	0	–	–	–	–


## Discussion

By using next-generation sequencing we analyzed 39 ALS-associated genes in a German cohort of both familial and sporadic ALS patients. In total, we detected 54 rare variants in approved disease genes and possible candidate genes, risk factors, and modifiers (synonymous variants excluded) in 48 patients which represents 60% of our total cohort.

We identified pathogenic or potentially pathogenic variants in 14 analyzed disease genes in 20 patients of whom 19 patients (95%) are affected by sporadic ALS. This finding is unexpected, as it demonstrates that a genetic background can actually be found in a major proportion of seemingly sporadic cases (25%; 19 of 74 patients with sALS). We also would have expected to find more variants in familial cases. Although guidelines and recommendations on how to evaluate unknown variants are published (see for example Richards et al., 2015), the assessment of the actual pathogenicity of detected unknown variants with regard to the patients’ phenotypes remains challenging and clear evidence on how a certain variant impairs the phenotype can only be achieved by extensive functional studies.

By focusing on possible candidate genes, risk factors, and modifiers, an interesting finding is the detection of heterozygous missense variants in the *GRN* gene in four patients affected by pure ALS (see **Table 2**). Three of the identified variants (p.V77I, p.V121M and p.A324T) are classified as being potentially pathogenic. Loss-of-function mutations in *GRN* are considered causative for frontotemporal lobar degeneration with ubiquitin-positive inclusions (Mackenzie et al., 2006). Recent evidence though suggests that missense mutations in *GRN* are also linked to the pathogenesis of ALS, especially as ALS and frontotemporal dysfunction are considered to represent a continuum of overlapping phenotypes, and a large proportion of ALS patients additionally experience frontotemporal dysfunction and vice versa (Sleegers et al., 2008; Cannon et al., 2013). Based on our findings, we recommend that *GRN* gene analysis should be included in routine molecular diagnostic settings and should also be considered in cases of pure ALS without frontotemporal involvement. Further, we detected seven missense variants in the *ITPR2* gene. Although Fernández-Santiago et al. (2011) as well as Chen et al. (2012) could not confirm an association of variants in *ITPR2* with ALS in a German and a Chinese cohort by SNP genotyping, we speculate that variation in the *ITPR2* gene could act as a modulating factor in ALS. A modulating effect might also exist for variants in *FGGY* (eight variants), *GRN* (four variants) and *UNC13A* (three variants).

These findings reflect the overall challenges in assessing the relevance of rare variants with respect to the phenotype as functional studies investigating the actual effect of these variants are largely missing. However, by the implementation of NGS in clinical genetics, we are now faced with increasing numbers of genes published as being possibly implicated in the pathogenesis of ALS. Such candidate genes gain further support from protein-protein interaction data. As rare variants in ALS associated genes according to current knowledge rather represent modifiers with effect on risk of developing the disease, age of onset, severity, or progression rate than disease causing mutations, further effort has to be made to understand how these modulating effects become evident in ALS. Investigating such modulating effects might lead to the identification of pathways that are not yet linked to ALS, enhancing our knowledge of ALS pathogenesis and higher-level neurodegenerative processes.

By performing repeat length analysis we identified five sporadic patients (6.25%) carrying pathogenic repeat expansions in the *C9orf72* gene. This is in line with Majounie et al. (2012) who reported on 5.2% of *C9orf72* repeat expansion carriers amongst German ALS patients. In two carriers of a pathogenic repeat expansion, we detected additional variants in ALS-associated genes. Although van Blitterswijk et al. (2012) suggested that additional genetic factors contribute to ALS pathogenesis in some carriers of a pathogenic *C9orf72* repeat expansion, we cannot assess the impact of additional variants on the patients’ phenotypes in our cohort study. We identified four further patients carrying intermediate length repeat expansions. According to recent literature, these might be pathogenic in ALS as patients carrying 20–29 repeats are phenotypically similar to those with more than 30 repeats (Byrne et al., 2014). However, as intermediate length repeats have been detected in both patients and healthy controls, their actual pathogenicity still remains unclear (Rohrer et al., 2015). Of the four individuals with intermediate length repeat expansions, two patients carried additional variants in disease related genes. In our cohort, patients with intermediate length repeats had an earlier age of onset than carriers of a pathogenic repeat expansion (averages of 57.0 and 67.6 years, respectively). This counter-intuitive result leads us to speculate that age of onset was not primarily influenced by the length of repeat expansions but possibly by other factors such as additional variants in other genes. However, we cannot draw a firm conclusion due to our limited cohort size. Surprisingly, we did not detect pathogenic repeat expansions in any of the familial cases, although this might also be because of the small sample size.

To evaluate the hypothesis that ALS might be of complex genetic origin, we searched our cohort for patients carrying more than one potentially disease-causing variant. We found that 15 patients (18.8% of our cohort, synonymous variants excluded) carry two or more variants in ALS-associated genes and that four of these 15 patients additionally carry an expanded or intermediate *C9orf72* repeat expansion. According to current findings, a complex model of inheritance is used to explain phenomena like reduced penetrance or even intrafamilial phenotypic variability. A hypothesis by Cady et al. (2015) for example implies that disease onset is influenced by the burden of rare variants in ALS-associated genes. The authors reported that 3.8% of 391 study participants harbored two or more variants in 17 analyzed disease genes and that these individuals had disease onset 10 years earlier than patients carrying only one variant. The considerable difference in percentage of patients carrying two or more variants (3.8% in Cady et al., 2015 vs. 18.8% in this study) might be explained by the fact that we included not only variants in approved disease genes but also in candidate genes, modifiers, and risk factors. In contrast, Cirulli et al. (2015) did not report an effect of the number of variants on the age of onset in their cohort of 2869 ALS patients and 6405 controls, but they do not draw a strong conclusion as they did not test for pathogenic *C9orf72* repeat expansions. In our data, we do see a later age of onset in patients carrying two or more variants. However, due to our smaller sample size, we cannot make statistically significant observations on a possible correlation and we cannot exclude that co-occurrence of multiple variants might have a disadvantageous effect on disease onset, severity, disease duration, or site of onset by affecting disease causing variants. As an example, the identification of *ITPR2* variants in co-occurrence in seven patients might hint at a possible negative effect of additional variants in the *ITPR2* gene. Further studies should include both next-generation sequencing and tests for pathogenic repeat expansion in a large cohort to resolve this open question.

To genetically and mechanistically link ALS to other pathologies of the NDD spectrum, we searched our cohort for potentially pathogenic variants in 238 genes that are associated with overlapping phenotypes and are covered by our diagnostic panel.

We identified potentially pathogenic variants in neurodegeneration-related genes in 12 patients. Although compound-heterozygosity for the detected variants in *GBE1* in pat #38 is not proven, we speculate that both variants might be at least concurrently causative, especially as the patient revealed UMN-dominant ALS, cognitive impairment, and progressive non-fluent aphasia (PNFA) upon his last clinical examination in 2012. GBE1 is a glycogen branching enzyme which is involved in glycogen synthesis. According to Ngo and Steyn (2015), there is a link between the selective degeneration of neurons in ALS and metabolic alterations: Deficits caused by decreased glucose metabolism may trigger hyperexcitability and subsequent selective degeneration of upper and lower motor neurons. Although the underlying mechanisms are still unclear, Wang et al. (2015) could show that the FUS protein (juvenile ALS) interacts to a great extent with mitochondrial enzymes and proteins involved in glucose metabolism. With regard to these presumptions, we speculate that pathogenic variants in *GBE1* might be causative for ALS or motor neuron degeneration, and that metabolic processes and involved genes must be taken into account in ALS genetics.

We detected known heterozygous variants in *SPG7* (paraplegin) in four patients. Recent evidence suggests that mutations in *SPG7* might be relevant in PLS as Mitsumoto et al. (2015) reported on the identification of a pathogenic heterozygous variant in *SPG7* in a patient affected by PLS. Paraplegin is part of the metalloprotease AAA complex, an ATP-dependent proteolytic complex located on the inner mitochondrial membranes, and functions in controlling protein quality and ribosomal assembly. Ferreirinha et al. (2004) showed that paraplegin-deficient mice develop axonal swellings as a consequence of accumulation of mitochondria and neurofilaments in the spinal cord which precedes axonal degeneration by impaired anterograde axonal transport. Although further studies are needed to assess the functional role of *SPG7* in human motor neurons, these findings hint at an important role of *SPG7* in motor neuron survival and support our hypothesis, that paraplegin is implicated in the pathogenesis of ALS and those pathogenic mutations in *SPG7* must be taken into account regarding genetic testing in ALS.

In summary, our results support recent observations whereby a genetic background is implicated in the sporadic form of ALS to a higher extent than assumed so far, and strengthen the upcoming hypothesis of ALS being a distinct manifestation of higher-level neurodegenerative processes rather than representing a discrete entity. Further, our results contribute to current discussions on a possible pathogenicity of intermediate repeat expansion in the *C9orf72* gene, especially in the interplay with additional variants in other ALS associated genes. In contrast to previously published studies, we could not prove an earlier age of disease onset in patients carrying multiple variants but speculate that variants in the *ITPR2* gene might act as a modulating factor in ALS. Additionally, our results lead us to assume that variants in *GRN* and *SPG7* might be implicated in the pathogenesis of ALS which is in line with the aforementioned hypothesis of common neurodegenerative processes leading to distinct phenotypes. Surprisingly, we did not detect clearly pathogenic variants in *SOD1* in our cohort, even though this gene is supposed to have a high impact on disease, encouraging us to launch a debate on the actual significance of *SOD1* in Germany.

## Conclusion

We investigated 39 ALS-associated genes in a German cohort of 80 familial and sporadic ALS patients utilizing next-generation sequencing. We identified 22 variants in disease-causing genes in 20 patients and additionally 32 variants in candidate genes, risk factors, and modifiers in 28 patients. Thus we detected variants in ALS-associated genes in 60% of our study participants, of whom the vast majority are sporadic cases. Surprisingly, pathogenic repeat expansions in *C9orf72* and potentially pathogenic variants in *SOD1* were both detected at lower frequencies than expected. Instead we identified potentially pathogenic variants in the *GRN* gene in four patients, indicating that the impact of *GRN* mutations is not limited to ALS-FTD and might account for pure ALS, too.

Furthermore, our cohort enabled us to evaluate the hypotheses that ALS is of complex genetic origin. According to this hypothesis, numerous variants have some degree of influence on the clinical phenotype caused by the pathogenic mutation. We did in fact identify patients carrying variants in more than one ALS-associated gene. In contrast to other studies, however, our results do not show that patients with multiple variants have an earlier age of onset.

As ALS should be seen in the context of wider neurodegenerative disorders, we investigated our cohort for potentially pathogenic variants in 238 neurodegeneration related genes. The most interesting findings are the identification of two variants in the *GBE1* gene that might be causative in a patient with UMN-dominant ALS and the detection of heterozygous variants in *SPG7* in four ALS patients. These findings would benefit from extensive high-throughput sequencing in large patient and control cohorts of different ethnic background in order to more accurately assess the overall variability in ALS-associated genes and to better evaluate their impact on the disease.

Our results support the notion that next-generation sequencing could help uncover the genetic heterogeneous basis of ALS and thus argue for the broader application of NGS techniques in routine diagnostic settings. Therefore, our results are of immediate relevance for clinical genetics as we recommend that genetic testing in German patients should be offered not only to those with familial ALS but also to those with apparently sporadic ALS. We propose a two-stage strategy starting with a *C9orf72* repeat analysis, followed by comprehensive gene panel sequencing if *C9orf72* negative. To meet the high number of possible differential diagnoses that mimic ALS, genes causing FTD, HSP, spinal muscular atrophy (SMA) and other entities that impair motor neuron function should be included. Whereas Sanger sequencing focused on a few commonly affected genes such as *SOD1*, panel sequencing offers the opportunity to cover all disease-associated genes in only one approach and thus reveals the genetic heterogeneity of ALS and increases detection rates. Additionally, panel sequencing allows for the detection of multiple variants acting on the individual phenotype which might enable statements for example on disease progression or severity. We hope that our results will contribute to deeper knowledge which will allow the identification of new therapeutic targets for example by interfering with distinct pathways or personalized therapeutic approaches in the future.

It was our aim to broaden the genetic landscape of ALS. We detected previously identified ALS-causing mutations, novel variants within recognized disease-causing genes and candidate genes, in addition to modifiers and risk factors. Assessing the impact of newly detected variants and their potential contribution to the ALS phenotype requires further investigation in order to determine their functional relevance. For several patients who gave their informed consent, we collected fibroblasts to provide the basis for the necessary functional work up.

## Author Contributions

Study concept and design: SK, MS, JP, and SB. Acquisition of clinical data and blood sample collection: JP and TGr. Analysis and interpretation of genetic data: SK, FB, AS, and MM. Drafting of manuscript: SK. Critical revision of manuscript: SK, FB, AS, MM, MS, LS, TGa, TGr, JP, and SB.

## Conflict of Interest Statement

The authors declare that the research was conducted in the absence of any commercial or financial relationships that could be construed as a potential conflict of interest.

## Footnotes

1 http://alsod.iop.kcl.ac.uk/home.aspx

2 http://string-db.org/
