# Supplementary material for: Rare Variants in Neurodegeneration Associated Genes Revealed by Targeted Panel Sequencing in a German ALS Cohort
Source: Front Mol Neurosci. 2016 Oct 13;9:92. doi: 10.3389/fnmol.2016.00092 (PMC5061735; doi:10.3389/fnmol.2016.00092)
Supplement: Supplementary file 1 [file Data_Sheet_1.DOCX]

**Supplement**

NDD panel genes:

We list HGNC symbols approved at the time of panel design. Updated current hgnc approved symbols are given in square brackets where appropriate.

*ABCA7, ABCB1, ABHD12, ACE, ACO1, ADAM10, ADARB1, AKT1, ALAD, APOE, APP, AR, ARHGEF7, ARHGEF9, ARSA, ATM, ATN1, ATP13A2, ATP1A3, ATP7B, ATXN3, B4GALT6, BDNF, BIN1, BSCL2, BST1, C10orf2, C19orf12, CACNA1A, CCR2, CCR3, CCR4, CD200, CD22, CD2AP, CD33, CD47, CDC42, CHRNB2, CLDN1, CLDN10, CLDN11, CLDN12, CLDN14, CLDN15, CLDN16, CLDN17, CLDN18, CLDN19, CLDN2, CLDN20, CLDN22, CLDN23, CLDN3, CLDN4, CLDN5, CLDN6, CLDN7, CLDN8, CLDN9, CLN3, CLU, CNR1, CNTF, CNTN4, CP, CR1, CSNK1G3, CST3, CXCL1, CYP2D6, DAPK1, DCAF17, DISC1, DNM1, DYNC1H1, EFEMP1, EIF2AK3, EIF4G1, EN1, EPHA1, EPO, EPOR, EVL, EXOC3L2, FA2H, FAS, FBXO7, FHL5, FKBP4, FOXA2, FTH1, FTL, FUCA1, FXN, GAB2, GAK, GARS, GBA, GBE1, GCDH, GCH1, GDNF, GJC2, GRIA2, GRIN2A, GSK3B, HEPH, HEXB, HIPK4, HMGCR, HMOX1, HPRT1, HSPA1A, HTRA2, HTT [SLC6A4], ICAM5, IFNG, IFNK, IL10, IL1A, IL1B, IL6, IL8 [CXCL8], IREB2, IRF4, JAK2, JPH3, KDR, KIAA0196, KIAA0226 [RUBCN], LOX, LRRK2, MAOB, MAPT, MECP2, MFN2, MOBKL2B [MOB3B], MOBP, MS4A6A, MTHFD1, MTHFR, MTT [MT-TP]P, NCAM1, NOTCH3, NPC1, NPC2, NT5C1A, NUCKS1, OCLN, OGG1, OPA1, OPA3, OTC, PANK2, PARK2, PARK7, PCDH11X, PDE8B, PDXK, PGK1, PICALM, PINK1, PITX3, PLA2G6, PLP1, PM20D1, POLG, POLG2, PPHLN1, PRKRA, PRNP, PSEN1, PSEN2, PVR, RAB7L1 [RAB29], RBMS1, RET, RRM2B, SCN7A, SELL, SEMA6A, SGCE, SLC11A2, SLC16A2, SLC25A4, SLC2A1, SLC39A11, SLC6A3, SMN1 (dup1), SMN1 (dup2), SMN2 (dup1), SMN2 (dup2), SMPD1, SNCA, SNCB, SNCG, SORL1, SPG20, SPG21, SPG7, SRGAP3, STX6, SUSD1, SYN1, SYNE1, TAF1, TBP, TF, TFR2, TFRC, TGFB1, TH, THAP1, TIMM8A, TJP1, TJP2, TJP3, TLR2, TLR4, TLR5, TMEM106B, TMEM126A, TNF, TOMM40, TOR1A, TP53, TYR, UBAP1, UBQLN1, VDR, VPS13A, VPS13C, VPS35, WFS1, ZFP64, ZFYVE26, ZNF746.*
